# Physician Behavior under Prospective Payment Schemes—Evidence from Artefactual Field and Lab Experiments

**DOI:** 10.3390/ijerph17155540

**Published:** 2020-07-31

**Authors:** Simon Reif, Lucas Hafner, Michael Seebauer

**Affiliations:** 1 Department of Economics, University of Erlangen-Nuremberg, Findelgasse 7, 90402 Nürnberg, Germany; lucas.hafner@fau.de (L.H.); michael.seebauer@fau.de (M.S.); 2 RWI—Leibniz Institute for Economic Research, Hohenzollernstr. 1-3, 45128 Essen, Germany

**Keywords:** health economic experiment, framing, physician behavior, prospective payment schemes, C91, C93, I11, I18

## Abstract

Recent experimental studies analyze the behavior of physicians towards patients and find that physicians care for their own profit as well as patient benefit. In this paper, we extend the experimental analysis of the physician decision problem by adding a third party which represents the health insurance that finances medical service provision under a prospective payment scheme. Our results show that physicians take into account the payoffs of the third party, which can lead to underprovision of medical care. We conduct a laboratory experiment in neutral as well as in medical framing using students and medical doctors as subjects. Subjects in the medically framed experiments behave weakly and are more patient orientated in contrast to neutral framing. A sample of medical doctors exhibits comparable behavior to students with medical framing.

## 1. Introduction

Understanding physicians’ reactions to incentives is crucial for the design of health care markets. In the seminal theory works on physician behavior of Arrow [1] and later McGuire [2], physicians are modeled to face a trade-off between personal profit and patient health. Following these early theoretical approaches, this trade-off has since been analyzed in empirical research (see Chandra et al. [3] for an introduction). Recent studies show that physicians perform more invasive treatments if this increases reimbursement [4,5] and increase consultation frequency not to improve treatment quality but to increase reimbursement [6]. There is also a growing amount of literature on experiments in health economics which shows that subjects playing the physician’s role care for patients to different extents [7,8,9,10,11,12].

Seminal laboratory experiments on physician behavior focus on settings, where physicians face a trade-off between their own payoff and patient well-being (see for example Hennig-Schmidt et al. [7], Brosig-Koch et al. [13], and Di Guida et al. [14]). These experiments are mainly conducted using medical framing and student subject pools. In this paper, we contribute to the growing experimental literature on physician behavior in three ways: First, we extend the physician decision problem by adding an insurer that finances medical service provision. Second, we conduct our laboratory experiment in neutral as well as medical framing to identify behavioral effects of contextual framing. Third, our subject pool consists of students as well as medical doctors which allows us to analyze whether professional experience in the relevant area influences decisions in the lab.

The division between receivers of services (patients) and those who pay for it (usually a health insurance) is an important feature of many health care systems. Such a third party that finances medical care does not only influence patients’ demand for services (most notably through moral hazard), but also the quality and quantity of care physicians supply. In their model of physician behavior, Chandra and Skinner [15] assume that medical care—although not payed for by the patient—is always subject to constraints, for example, to a lack of resources or ethical norms against spending too many resources. A qualitative study by Hassell et al. [16], a survey by Tilburt et al. [17], and a discrete choice experiment by Pedersen et al. [18] all indicate that physicians take into account the costliness of their services and the scarcity of available resources for treating patients. We analyze physician behavior when an insurer finances medical service provision. This extends the seminal works of Hennig-Schmidt et al. [7] and Brosig-Koch et al. [13] which focus on the bilateral relationship between physicians and patients, in particular with respect to physician payment schemes. We add an insurer that provides a budget for treatment. Such a change can influence individual decisions in experiments as the number of affected agents increases [19,20]. We model the financing of medical care as a prospective payment scheme (PPS) where the budget physicians can spend is determined by the patient’s diagnosis. In most PPS, physicians report patient type by ICD-codes and receive a budget based on a classification algorithm ([21], p. 43ff.). This is not the only common way to organize hospital reimbursement, and physician behavior under PPS has also been subject to many economic studies (e.g., Davis and Rhodes [22], Moreno-Serra and Wagstaff [23], Cutler [24]). The evidence is accruing that physicians over-report patient severity under PPS in order to increase reimbursement (see results from administrative data in Dafny [25], Silverman and Skinner [26], Jürges and Köberlein [27], Fang and Gong [28], Reif et al. [29], and the recent laboratory experiment by Hennig-Schmidt et al. [30]). There is however mixed evidence on whether the extra reimbursement is used to improve care, enrich the physician, or both. We contribute to the literature by modeling the patient–physician–insurance relationship with a PPS to analyze physician behavior in such a more complex decision problem. More specifically, our physicians observe a patient’s medical needs and report the severity of the patient’s sickness to the insurer. The insurer then provides a budget for treatment dependent on the reported diagnosis. In turn, this budget can be spent by the physician to provide medical care to the patient. As the physician is the only agent that makes decisions in our setting, we technically implement a three-person dictator game. Among the first to implement such a design were Charness and Rabin [31] as well as Engelmann and Strobel [32] who find that dictators want to avoid extreme inequality among the subjects. In a meta-study on dictator games, Engel [33] finds that dictators in total give more if there is more than one recipient.

The choices of subject pool and framing are crucial for the design of economic experiments. In studies that analyze physician behavior, the most common choice is a student subject pool with medical framing (See, for example, Lagarde and Blaauw [34], Brosig-Koch et al. [9], Brosig-Koch et al. [35], Brosig-Koch et al. [13], Keser et al. [10], Hennig-Schmidt et al. [7], and Kairies and Krieger [36]). Abbink and Hennig-Schmidt [37] and Gneezy et al. [38] emphasize that contextual framing has advantages as well as disadvantages, and therefore the framing choice depends on the underlying question. In particular for studies on physician behavior, framing might induce experimental subjects to behave as they *expect* physicians to behave [39]. However, neutral framings might induce varying contexts in the subjects’ mind, which can affect decisions but are unobservable to the researcher. For example, Kimbrough and Vostroknutov [40] find that individual norms correlate with pro-social behavior and Kesternich et al. [41] show in an experiment with medical students that changing perceived context by inducing professional norms influences how subjects distribute stakes between group members. We therefore want to explicitly analyze how changing context framing affects behavior in laboratory experiments. When it comes to choosing a subject pool, Harrison and List [42] suggest that not only students but also professionals should take part in experimental studies. The results from Brosig-Koch et al. [35] show that in the experimental analysis of physician behavior the decisions of business and economics students are similar to those of medical doctors. In contrast, Wang et al. [43] find that medical doctor subjects provide less patient benefit. In general, Engel [33] shows that non-student subjects give more in dictator games. We contribute to both the discussion on framing as well as on subject pool by conducting our experiment with three different subject pools: a student sample in neutral framing, a student sample with medical framing, as well as a sample of medical doctors with medical framing.

We find that physicians trade-off between their own payoff and patient utilities as well as the payoff of the third party. Additionally, we show that concern for patients is higher when the experiment is framed in a medical context. Our results also suggest that medical doctors behave similar to students in laboratory experiments.

The remainder of the paper is structured as follows. In the next Section 2, we introduce our experimental design. The results from our experiments are presented in Section 3, and in the final part Section 4 we conclude.

## 2. Experimental Design

We conduct artefactual field and lab experiments to analyze physician behavior. *Physicians* observe the medical needs of a *Patient*, report the severity of his/her illness to an *Insurer*, and use the budget from the *Insurer* to provide Medical Services to the *Patient*. The third party that finances the Medical Service provision and the related reporting stage are the two main design extensions to the seminal works of Hennig-Schmidt et al. [7] and Brosig-Koch et al. [13], where physicians observe patient severity and then directly provide Medical Services.

### 2.1. Framing and Subject Pool

In order to identify behavioral effects of framing, we conduct our experiment in a neutral setting as well as in a setting with medical context. Subjects in our experiment take on the roles of either *Patients*, *Physicians*, or *Insurer*. Naming of participant types varies between neutral and medical framing. We call them *Participant A*, *B*, and *C* in the neutral framing, whereas in the medical framing we call them *Patient*, *Physician*, and *Insurer*, respectively. The framing does not influence the underlying mechanism of the experiment. Therefore, for ease of readability, we will use the medical terms to describe the experimental design. Subjects in our experiment were a sample of students as well as a sample of medical doctors. This allows us to analyze whether professional experience in the relevant area influences the decisions in the lab.

### 2.2. Group Composition and Roles

At the beginning of each experimental session, we divide subjects randomly and anonymously into groups of three. The group composition remains unchanged throughout the whole experiment. Subjects do not know the other two group members but they know that the composition of the groups will not change during the experiment. There is no interaction across groups, thus the outcomes of the members of one group only depend on the decisions of the members within this group.

Only the *Physician* makes decisions that can influence his/her own payoff and determines the payoff of the other participants within their group. *Patient* and *Insurer* will not make any decisions in the experiment (Hennig-Schmidt et al. [7] and Brosig-Koch et al. [13] operationalize patient needs by giving the patient payoff to charity. While this approach has many advantages, it is less suitable in our setting. It would not make much sense to use charity giving for the insurance and consequently, if only the *Patient* would be represented by donations to charity, *Physician* behavior can be influenced by whether they prefer donating to a charity or to another subject in the lab. In order to avoid such incentives, we have all three group members represented by subjects in our lab.).

First, every participant makes decisions, as if he/she was in the role of the *Physician*. After all participants made their decisions, we announce the random assignment of the participants to the roles of *Patient*, *Physician*, and *Insurer*. Only the decisions of the group member who is assigned to the role of the *Physician* are payoff relevant for the members of the respective group. The decisions of the participants who are assigned to the roles of *Patient* or *Insurer* are irrelevant for the group members (Even though the decisions of the participants who are assigned to the roles of *Patient* or *Insurer* are irrelevant for the final payoff of their respective group members, we can use their decisions in a strategy method sense for our analysis of *Physician* behavior. Although this form of strategy method might itself increase pro-social behavior, it is an unproblematic choice in our setting as we have no reason to believe that the effect of the strategy method differs by treatment. Further, Brandts and Charness [44] show in their survey article that treatment effects obtained from strategy method experiments are also obtained using direct-response designs.).

### 2.3. Relationship between the Group Members

**Figure 1:**
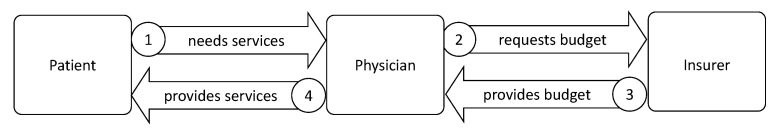
Relationship between the group members.

*Physicians* have to provide Medical Services to the *Patients*. The provision of Medical Services is associated with costs and, in order to cover costs, the *Physician* has to request a budget from the *Insurer*. *Physicians* request a budget by reporting information about the *Patient* to the *Insurer* and the reported information determines the size of the budget. Subsequently, the *Physician* decides on how many Medical Services he/she wants to provide to the *Patient*. Figure 1 illustrates the relationship between the group members.

### 2.4. Roles and Payoffs

We will now, step by step, introduce the three roles (*Patient*, *Physician*, and *Insurer*) in detail.

#### 2.4.1. Patient

Every *Patient’s* payoff can either be 0 or 90 Taler (our experimental currency). The *Physician’s* decision on the number of Medical Services provided determines the probability to earn 90 Taler. We implemented a probabilistic relationship between *Patient* payoff and *Physician’s* decision, as we consider it to be more realistic than a deterministic relationship. In reality, the health outcome of a *Patient* is influenced by the *Physician’s* decision to a great extent. However, other factors can also have an influence (e.g., the predisposition of a *Patient* or the effectiveness of prescribed drugs), in order to ensure in our experimental design that sickness of the *Patient* after a medical intervention cannot unambiguously be traced back to misbehavior of the *Physician*. Huck et al. [45] use a similar mechanism and Martinsson and Persson [46] show that *Physicians’* decisions are similar in a probabilistic and in a deterministic setting.

The severity of a *Patient’s* illness can influence *Physician* behavior [9,29]. In order to allow for such heterogeneity in our experiment, we introduce three types of *Patients*—low type (L), medium type (M), and high type (H)—which represent different severities of *Patient’s* illness.

The three types of *Patients* need different numbers of Medical Services in order to maximize their probability of receiving the payoff of 90 Taler. The highest probability of receiving a payoff is 95%. Two units of Medical Services are optimal for L type *Patients*, whereas M type *Patients* need four and H type *Patients* six units. Providing too many Medical Services is equally harmful for the *Patient* as providing too few Medical Services. The probability to earn 90 Taler is reduced to 65%/35%/5%, when the number of Medical Services provided is one/two/three or more unit(s) above or below the optimum, respectively.

Table 1 shows the *Patient* type-specific connection between number of provided Medical Services and the probability to earn 90 Taler.

At the end of the experiment, the *Patient* learns about his/her final payoff. The *Patient* does not learn about his/her type or the number of Medical Services provided by the *Physician*. We consider this design choice to be a realistic representation of actual doctor–patient relationships where asymmetric information is present. The *Patient* does not make any decisions in the experiment.

#### 2.4.2. Physician

Every *Physician* faces the task to provide Medical Services to each *Patient* type, L, M, and H, consecutively (In each treatment, one-third of the subjects faced the sequence L-H-M, M-L-H, and H-M-L, respectively. To ensure comparability across treatments, we kept this sequence pattern constant for all subjects in all experimental sessions.). The different *Patient* types are independent—the provision of services to one *Patient* has no effect on the budget or Medical Service provision of another *Patient*. The potential number of Medical Services provided is an integer between one and six and is associated with costs. Every unit of Medical Services provided costs 15 Taler. In Table 2, we give an overview on the potential number of Medical Services and the associated costs.

In order to cover costs, the *Physician* has to request a budget by reporting a *Patient* type to the *Insurer* (see decision screen on Figure A2). The *Physician* can report any type of *Patient* (L, M, or H) independently of the true type of the *Patient*. Therefore, it is possible to report false information—which we call misreporting (Two recent papers, Hennig-Schmidt et al. [30] and Groß et al. [47], provide an experimental investigation specifically on misreporting by physicians.). Two kinds of misreporting are possible—overreporting and underreporting. Overreporting (underreporting) refers to the case where the *Physician* reports a higher (lower) type than the true *Patient* type to the *Insurer*. An example of overreporting would be if the true *Patient* type is L whereas the reported type is M. We summarize the possible reporting behavior of the *Physician* in Table 3.

The reported *Patient* type determines the assignment to a budget group and therefore the size of the budget, comparable to diagnosis related groups in PPS.

*Physician* payoff can either be determined by a *fee for service* payment system (FFS) where the number of Medical Services provided determine the payoff of the *Physician* or a *capitation* payment system (CAP), where the payoff of the *Physician* is independent of the number of Medical Services provided. Under FFS, the *Physician* receives 15 Taler per unit of Medical Service provided. Under CAP he/she receives 50 Taler in any situation. We present an overview of the two *Physician* payment systems in Table 4. FFS represents the case where the *Physician* acts on his/her own bill, while in CAP the *Physician* receives a fixed wage from a hospital.

#### 2.4.3. Insurer

One participant in the experiment represents the *Insurer*, which is endowed with 130 Taler for each *Patient* in all experimental conditions. Dependent on the *Patient* type reported by the *Physician*, the budget for the *Physician* is withdrawn from the endowment of the *Insurer*. We implement a budget scheme with two groups, where type L and M *Patients* are assigned to budget group I (45 Taler), which is sufficient to cover costs for the average *Patient* of type L or M. This design feature reflects a crucial aspect of many PPS, namely, that costs for an average *Patient* are reimbursed, and consequently not enough budget is available if *Patient* severity is at the upper end of a budget group definition. If an H type *Patient* is reported, budget group II (90 Taler) is provided, which covers the cost for optimal Medical Service provision. In case the budget is not fully spent (*Physician* reports L/M/H and provides less than 2/4/6 Medical Services), the unused budget benefits none of the three group members. This is comparable to actual PPS, where “unused” budget benefits the hospital in general, but not the physician in charge or the insurer. With this design choice, we shut down the possibility for the *Physician* to cross subsidize between *Patients*, where medical service provision choices could be influenced by endogenous preferences towards low or high severity *Patients* (Actually, PPS are designed in a way where hospitals make profits on some patients and losses on others, and are thus incentivized to reduce average costs. There is also a feedback mechanism which adjusts future budgets to actual average costs that could result in strategic reporting. To keep our experimental design as simple as possible, we leave these channels to future research.).

Table 5 summarizes the information of the budget groups and available budgets dependent on the reported *Patient* type.

### 2.5. Physician Decision Problem and Conjectures

In total, we implemented six treatments with different combinations of our experimental variations. Table 6 shows an overview of our treatments including their respective abbreviation and the number of subjects in each treatment.

An overview of the potential payoffs of *Physicians* and *Insurer*, as well as the expected payoffs of the *Patient*, is given in Table 7.

We implemented three forms of experimental variation: First, we vary the *Physician* payment system, which is either dependent (fee for service) or independent (capitation) of the provision behavior of the *Physician*. This is our baseline variation which is closely related to the previous literature. Second, we use two different types of framing: one introducing a medical context and a neutral one without context. Third, we vary the subject pool, where participants of the experiment are either medical doctors or students.

We now derive conjectures about the behavior of the *Physician* under our three experimental variations. In all treatments, the payoffs for all three group members are solely determined by the *Physician*. Reported *Patient* type is the only factor that affects the payoff for the *Insurer*, as the assigned budget is subtracted from its initial endowment. The Payoff of the *Insurer* is therefore independent of subsequent Medical Service provision. Although only the provision of Medical Services affects *Physician’s* and *Patient’s* payoffs, the preceding reporting decision plays an indirect role for their payoffs by the possible restriction to the number of affordable Medical Services.

Ultimately, the decisions of the *Physician* depend on how he/she values the well-being of all three group members. Generally, if he/she attaches a high value to the Payoff of the *Insurer*, he/she reports a low *Patient* type. If he/she however attaches a high value to the *Patient* payoff, he/she reports a type such that the provided budget is sufficient for the optimal number of Medical Services. In the capitation system, the *Physician* only faces the possible trade-off between *Insurer* and *Patient* payoff. In contrast, in the fee for service system he/she also influences her own payoff. If he/she attaches a high value to his/her own payoff he/she will report a high *Patient* type and subsequently provide a high number of Medical Services. As our *Physician* payment system induces different personal incentives, we expect participants to behave differently across fee for service and capitation systems. Following the theoretical predictions in Ellis and McGuire [48] and findings from previous health economic experiments [7,11], we expect more overreporting and overprovision of Medical Services in the fee for service system. We do, however, not expect the different payment systems to affect the *Physician’s* preferences towards either *Insurer* or *Patient* payoff (Differences in the reporting and provision behavior in fee for service in contrast to capitation systems that favor either *Insurer* or *Patient* payoff can be explained by the presence or absence of own pecuniary incentives of the *Physician*.).

**Conjecture** **1.** *A fee for service Physician payment system leads ceteris paribus to overprovision of Medical Services, compared to a capitation payment system.*

Our second experimental variation affects the presentation of our experimental setting, which is framed either in a neutral or a medical way. In the neutral framing, subjects either face a trade-off between “Participant A” and “Participant C” (capitation) or their own payoff as well as the payoff of “Participant A” and “Participant C” (fee for service). In the medical framing, subjects make decisions which can affect themselves, the *Patient*, and the *Insurer*. Findings of an earlier health economic experiment suggest that economics students “[...] allocate in less own payoff maximizing ways [...]” when they are in a medically framed setting ([39], p. 6). We therefore expect the decision of the *Physician* to be more *Patient*-oriented in the capitation case by introducing a medical framing compared to the neutral framing, as this is more in line with professional norms of *Physicians* whose main purpose is to restore the health of her *Patients*. In line with this, in the fee for service systems we expect that *Physicians* will behave less selfishly, which leads to lower harm for the *Patient* compared to neutrally framed fee for service systems.

**Conjecture** **2.** *The medical framing induces ceteris paribus more Patient-oriented behavior, while the neutral framing leads to more selfish, own payoff maximizing behavior.*

Our third experimental variation is the subject pool, which consists either of students or medical doctors. With this variation we can test whether the norms induced by the medical framing lead to behavioral differences between students without medical background and trained medical doctors. Brosig-Koch et al. [35] show that medical doctors behave in a similar way as students but are on average more concerned with *Patient* payoff, while Wang et al. [43] show that medical doctors are slightly less *Patient*-oriented. As the differences in these two studies were small we expect no difference between subject pools.

**Conjecture** **3.** *Medical doctors behave similarly compared to business and economics students.*

### 2.6. Experimental Protocol

Our computerized experiment was conducted at the Laboratory for Experimental Research (LERN) in Nuremberg, Germany. The experiment was programmed and conducted using z-Tree [49], and ORSEE [50] was used to recruit the student subjects. In total, 105 students and 21 medical doctors participated in our experiment (CNS, FNS—27 students; CMS—24 students; FMS—27 students; CMD—12 medical doctors; FMD—9 medical doctors. The sample size allows for a minimal detectable effect size (MDES) for α=0.1 and β=0.8 of 0.5 for the student samples and a MDES of 0.9 for the doctor sample. This is in the range of effect sizes reported in previous experiments on physician behavior.). The average age of our student sample is 23 years and the average age of our medical doctors is 38 years. We do not observe significant age differences across our treatments for students or medical doctors. In total, 13 medical doctors are male and 8 are female. Sixty-one students are female and while 44 are male.

Our student sample consists mainly of undergraduates in economics and business administration. The medical doctors were recruited at a teaching day of an advanced education program in management, which took place in the same building where our laboratory is located (Approximately 90% of our student sample consists of economics and business administration students and 10% are students in Engineering, Law, or study to become a teacher. A sample of doctors that are about to obtain a business degree is clearly not a representative sample of the doctor population. In the existing literature, real doctors behave in a more patient-oriented manner than students. Results from this selected sample are therefore giving a lower bound on the true effect.). We implemented a between subjects design—each subject participated in one treatment only and each treatment was conducted in a separate session. The experimental procedure was identical for all sessions. Upon arrival at the laboratory, subjects were randomly allocated to partitioned computer terminals and given hard copy instructions (Translated screen shots of the experiment as well as the translated instructions and the control questions can be found in the Appendix C, Appendix D, Appendix E, Appendix F and Appendix G. The original German materials are available upon request.). After having read the instructions, subjects had to answer a set of control questions.

The experiment did not begin until all subjects answered all questions correctly. When subjects revealed a lack of understanding, the experimenters explained the respective problem to them personally. Subjects could take as long as they needed to make decisions, to view result screens, and to complete the control questions. All subjects made their decisions in full anonymity.

Sessions lasted approximately one hour. Earnings were expressed in Taler which were exchanged for cash at the end of the session for 1 EUR per 10 Taler for the student subjects and 4 EUR per 10 Taler for the medical doctor subjects (These different exchange rates are comparable to the implementation of Brosig-Koch et al. [35]. Differences in exchange rates are implemented to account for different opportunity costs of different subject pools.). Student (medical doctor) subjects earned an average of 10.26 (44.57) EUR, including the show-up fee of 4 (16) EUR.

## 3. Results

In this section, we present the experimental results for physicians provision behavior. We compare the provision behavior across physician payment systems, framings, and subject pools. We continue with a regression analysis in order to compare conditional means of the payoffs for the group members. Results for the corresponding reporting decisions are presented in Appendix A.

### 3.1. Average Provision Behavior

**Figure 2:**
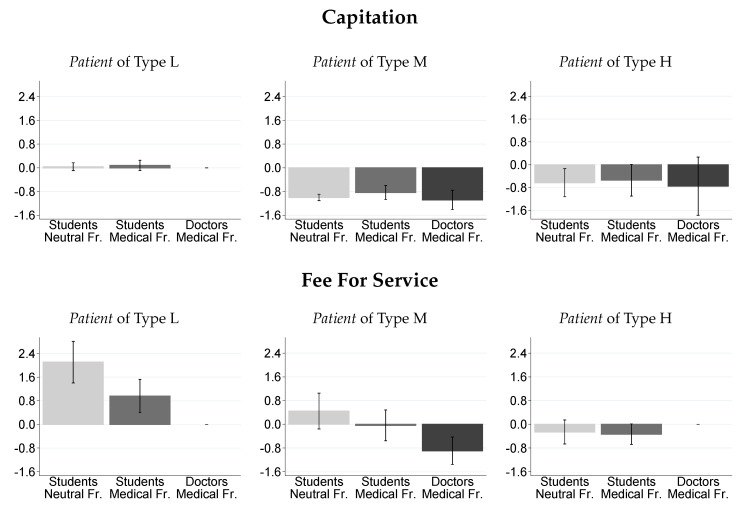
Average deviation from optimal treatment across experimental conditions. **Notes:** This figure illustrates average deviation from optimal treatment and 95% confidence intervals across experimental conditions. The values are standardized such that optimal Medical Service provision is 0; positive (negative) values indicate overprovision (underprovision). Each subject decided on the reporting and provision of (medical) services of every *Patient* type. Therefore, the total number of observations is 126. Number of observations in respective treatments: CNS—27, CMS—24, CMD—12, FNS—27, FMS—27, FMD—9. Table A7, Table A8 and Table A9 (in the Appendix B) contain the average deviation from optimal treatment and tests for significant differences.

**Figure A1:**
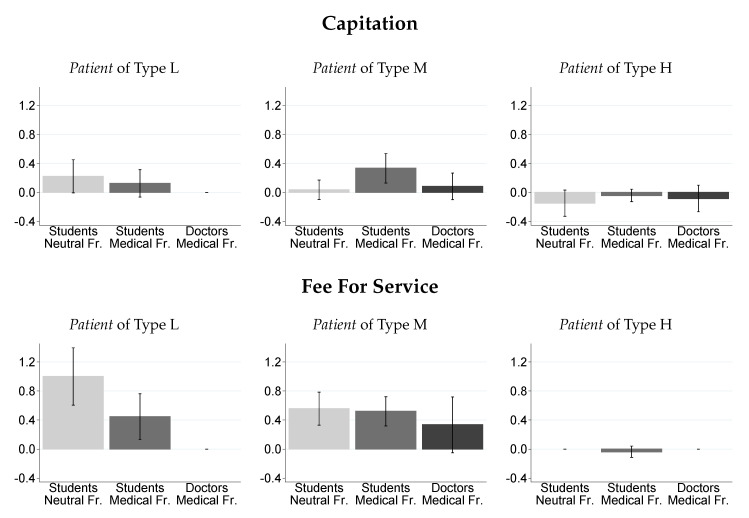
Average misreporting across experimental conditions. **Notes:** This figure illustrates average misreporting and 95% confidence intervals across experimental conditions. Misreporting refers to the case where the reported *Patient* type differs from the true *Patient* type. Positive misreporting corresponds to overreporting, while negative misreporting corresponds to underreporting. Each subject decided on the reporting and provision of (medical) services of every *Patient* type. Therefore, the total number of observations is 126. Number of observations in respective treatments: CNS—27, CMS—24, CMD—12, FNS—27, FMS—27, FMD—9. Table A4, Table A5 and Table A6 (in the Appendix B) contain the average misreporting values and tests for significant differences.

**Figure A2:**
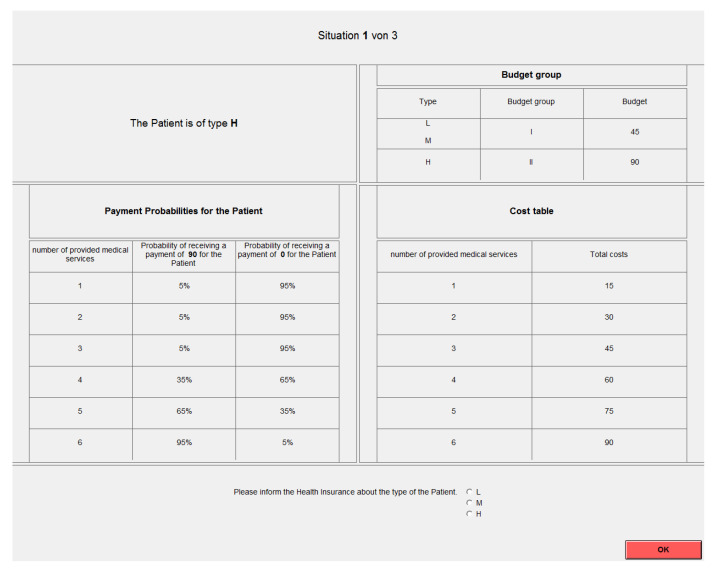
Reporting stage.

Figure 2 shows the average deviation from optimal Medical Service provision for each *Patient* type across the experimental treatments. Here, negative values indicate average underprovision, while positive values indicate average overprovision of Medical Services. As in Figure A1, the first row shows deviation from optimal treatment for the CAP and the second row for FFS. Columns indicate *Patient* types, and within each subfigure each bar represents students in the neutral framing, students in the medical framing, and doctors in the medical framing, respectively. In the CAP treatments, medical service provision is on average optimal for type L *Patients* and there is underprovision for M and H *Patients*, independent of subject pool and framing. For L *Patients* in the FFS treatments, there is most overprovision for students in the neutral framing and some overprovision for students in the medical framing. If we look at type M *Patients*, there is some overprovision for students in the neutral framing, on average optimal provision for students in the medical framing and underprovison for the doctor sample. H type *Patients* receive on average fewer than optimal Medical Services for both student samples and the optimal number of services in the doctor sample.

In the following subsections, we present the results in more detail by comparing average provision behavior across experimental conditions. For hypothesis testing, we use Mann–Whitney U tests. First, we focus on the difference between the physician payment systems (Section 3.2), second on the difference between neutral and medical framing (Section 3.3), and third on the difference between student and medical doctor subject pool (Section 3.4).

### 3.2. Differences between Fee For Service and Capitation

There are clear differences between the physician payment systems in the provision of Medical Services (Table 8). While there is barely any deviation from optimal Medical Service provision for type L *Patients* in the capitation systems, there is significant overprovision in the fee for service systems in the student samples (CNS 0.04 vs. FNS 2.11 and CMS 0.08 vs. FMS 0.96). Participants from our sample of medical doctors always provide the optimal number of Medical Services for type L *Patients*.

On average, medical service provision is lower than optimal for type M *Patients* in all experimental conditions apart from the neutrally framed student sample with fee for service. It is significantly lower for both student samples when the capitation physician payment system was implemented (CNS −1 vs. FNS 0.44 & CMS −0.83 vs. FMS −0.04. This indicates that type M *Patients* are better off in the fee for service system. However, when we look at the absolute deviations from the optimum, displayed in Table A11 in the Appendix B, we see that this is not the case, as there is both under- and overprovision of Medical Services.).

The pattern is similar for our sample of medical doctors, although the difference is not significantly different from zero (CMD −1.08 vs. FMD −0.89). When participants reported truthfully, the available budget is not sufficient to provide the optimal number of services for type M *Patients*. As many participants reported the true type of type M *Patients*, it is not surprising to observe high levels of underprovision for type M *Patients* in the capitation setting.

For type H *Patients* in the student samples, we observe significant underprovision of services in both payment systems. The underprovision is more pronounced in the capitation system, although the differences are only significantly different from zero in the comparison of the neutrally framed students (CNS −0.63 vs. FNS −0.26), while the difference is small and insignificant for medically framed students (CMS −0.54 vs. FMS −0.33) and insignificant for medical doctors (CMD −0.75 vs. FMD 0). Such underprovision neither benefits the *Physician* (who receive a fixed income under capitation) nor the *Insurer* as the budget is withdrawn independent of actual services provided. Harming the *Patient* by underproviding medical services can thus be seen as a choice that makes the (expected) payoff of all three participants more equal.

We find significant behavioral differences between capitation and fee for service systems, independent of *Patient* type and framing. Subjects in fee for service systems are more likely to overreport and overprovide for L and M type *Patients*, while they are less likely to underreport and underprovide for type H *Patients*. The overall effect sizes of the fee for service payment on medical service provision are 0.93 for students under neutral framing, 0.55 for students under medical framing, and 0.38 for our sample of doctors. Qualitatively, this is in line with the findings of Brosig-Koch et al. [13] and Brosig-Koch et al. [35], although the effect sizes are only half as large in our setting with a third party as well as a second decision stage in our experiment.

**Result** **1.** *The different physician payment systems have a significant influence on the reporting and provision behavior. The fee for service system induces more selfish Physician behavior in the student samples.*

### 3.3. Differences between Neutral and Medical Framing

When we compare the provision behavior between neutral framing and medical framing, the only significant difference we find is for type L *Patients* in the fee for service setting, where the overprovision of Medical Services is higher in the neutral framing (Table 9). This is in line with results from Kesternich et al. [41] who show that salience of professional norms increases pro-patient behavior of physicians. Our second overall result is therefore:

**Result** **2.** *The medical framing induces a slightly more Patient-oriented behavior of the Physicians.*

### 3.4. Differences between Student and Physician Samples

As a last comparison, we evaluate the effects of different subject pools by comparing the medically framed experiments of student and medical doctor subjects (Table 10). The provision of Medical Services also hardly differs between the subject groups. Again, the only significant difference is for type L *Patients* in the fee for service setting, where students overprovide significantly in contrast to the doctors who provide the optimal number of services. This is in line with results from Brosig-Koch et al. [35] who also find slightly more patient-oriented behavior for a sample of doctors compared to students. Our third overall result is therefore:

**Result** **3.** *Behavior of medical doctors and medically framed students is not significantly different for type M and H Patients. We do find a significant difference for type L Patients in Fee For Service, where Students behave more selfishly.*

### 3.5. Regression Analysis—Payoffs and Experimental Variations

Reporting and provision of Medical Services ultimately results in different payoffs for *Patient*, *Physician*, and *Insurer*. In order to analyze how the different experimental variations influence the trade-off between the participants, we conduct a regression analysis. Linear regression models allow us to identify differences in the conditional means of each experimental variation while keeping constant the other variations. As the payoffs of *Patient*, *Physician*, and *Insurer* are interdependent by design, we apply a seemingly unrelated regression model, to take the resulting cross equational error correlation into account ([51], pp. 333–335). Table 11 summarizes the regression results. The dependent variables of one set of seemingly unrelated regressions are the expected payoff of the *Patient*, the payoff of the *Physician*, and the remaining endowment of the *Insurer* (The actual payoff of the *Patient* is zero or 90. As the provision of Medical Services determines the probability of receiving a payoff, we use the expected payoff of the *Patient*.). We estimate separate sets of seemingly unrelated regressions for the different *Patient* types. As explanatory variables we use dummies for the variations in *Physician* payment systems (“Fee For Service”), type of framings (“Medical Framing”) and subject pools (“Medical Doctor”). (We also estimate the models controlling for subject characteristics. We control for age and gender of the subjects, as well as measures for risk preferences and social value orientation. This does only marginally influence the results for our student sample, while there are some differences for the *Physician* sample driven by the small sample size given the number of variables in the regression models (see Table A15 in the Appendix B).

In regressions with low and medium *Patient* types, the *Fee For Service* coefficient is negative and significant for the *Patient* and *Insurer*, indicating that the *Physicians* are willing to harm both other participants to increase her personal payoff. This is clearly visible for M type *Patients* where the *Fee For Service* coefficient for the *Physicians* is significantly positive. Whereas for L type *Patient*, the fixed payment under *Capitation* is comparably high such that *Fee For Service* does not induce a significant difference in the payoff of the *Physicians*. For H type *Patients*, we observe a higher *Patient* payoff in the *Fee For Service* setting. The payoff for the *Physicians* is also significantly higher in the *Fee For Service* setting for type H *Patients*, while the *Insurer* payoff is lower. In line with Result 1 in a *Fee For Service* physician payment system, we find more selfish behavior of the *Physicians* at the expense of *Patient* and *Insurer*.

When we look at the effects of different framings, we see that for type L *Patients*, a medical framing induces a higher payoff for both the *Patient* and the *Insurer*. For M type *Patients*, the payoff for the *Patient* is higher in the medical framing, while the *Insurer* payoff is lower. *Physician* payoff in medical framing is lower, however this difference is only statistically significant for L type *Patients*. For H type *Patients*, we find no significant effect of the medical framing on any of the three payoffs. This shows that—in line with Result 2—medical framing induces *Physicians* to behave in a more *Patient*-oriented manner, at their own cost and expense of the *Insurer*.

Looking at the different subject pools reveals only minor differences between students and medical doctors. The only significant differences are a higher *Patient* payoff for L type *Patients* and a higher *Insurer* payoff for M type *Patients* in the medical doctor sample. All other differences between subject pools are small and not significantly different from zero. This is also in line with Result 3, as students and medical doctors behave rather similar, with medical doctors caring slightly more about the *Patient* payoff.

## 4. Discussion and Conclusions

We conduct a controlled laboratory experiment to investigate how *Physicians* trade-off between their own, their *Patients’*, and the *Insurers’* benefits under prospective payment schemes. We modify the experimental design of the seminal works by Hennig-Schmidt et al. [7] and Brosig-Koch et al. [13] and introduce a third party that provides a budget for Medical Service provision. A further contribution to the literature is our variation of framings and subject pools.

Even though we introduce a third party in our experiment, our results on the differences between a capitation and a fee for service physician payment system are similar to other experimental studies. Capitation systems are more beneficial for *Patients* with a low severity of illness, while in fee for service systems, *Patients* with low severity of illness are harmed due to overprovision of Medical Services. For *Patients* with a high severity of illness, the fee for service system is more beneficial, as the personal financial incentive of the *Physician* to provide more services is aligned to the higher demand for Medical Services of those *Patients*.

In addition, we show that *Physicians* care about the payoff of a third party that finances medical service provision, an observation in line with results from surveys of physicians. This care for the third party can lead to underprovision of Medical Services to save costs for the third party. This is in particular the case where *Physicians* are not incentivized to provide many Medical Services. Previous experimental studies on physician behavior were not able to identify such concerns.

In our experiment, the behavior of participants is similar across framings and subject pools. Nevertheless, there are some differences. We find that neutrally framed experiments induce more selfish behavior, while participants in the medically framed experiments did care more about the *Patient* payoff. For our sample of medical doctors, we observe the most *Patient*-oriented behavior.

Our results show that direct financial incentives shape the behavior of *Physicians*. Nevertheless, distributional concerns regarding costs for the *Insurer* and well-being of the *Patients* play an important role in our controlled experiment. However, from an experimental research perspective, two main aspects of our paper need further investigation. First, the external validity of our experiment is limited. The patient–physician relationship is much more complex in the real world setting compared to our simplification. This simplification is especially important when it comes to patient payoff. In our experiment, patient well-being is represented by a stochastic payment to the participant. This modeling choice is a strong abstraction from actual patient well-being. Field experimental studies can provide a more externally valid assessment of medical care provision. Second, the health insurance in our experiment is represented by a single participant. Further research on efficiency and equality preferences within the pool of insured individuals is needed for a complete assessment of incentives and preferences in reimbursement schemes. From a policy perspective, further research on the interaction of *Physician* payment and budget provision is needed to improve current incentive structures in the medical sector.

## Figures and Tables

**Table 1:** *Patient* payoff probabilities in %.

		Number of Services Provided					
		1	2	3	4	5	6
	L	65	95	65	35	5	5
*Patient* Type	M	5	35	65	95	65	35
	H	5	5	5	35	65	95

**Notes: ***Patient’s* probability to earn 90 Taler for three types of *Patients* (low type (L), medium type (M) and high type (H)).

**Table 2:** Costs of provided services.

	Number of Services Provided					
	1	2	3	4	5	6
Costs	15	30	45	60	75	90

**Table 3:** Reporting options of the *Physician*.

		Reported *Patient* Type		
		L	M	H
	L	Truthful	Overreporting	Overreporting
**True *Patient* Type**	M	Underreporting	Truthful	Overreporting
	H	Underreporting	Underreporting	Truthful

**Table 4:** *Physician* payoff by service provision.

		Number of Services Provided					
		1	2	3	4	5	6
Payment System	Fee For Service	15	30	45	60	75	90
	Capitation	50	50	50	50	50	50

**Table 5:** Assignment of budget groups and costs for optimal number of services.

(Reported) Type	L	M	H
Costs for optimal service	30	60	90
Budget Group	I		II
Available Budget	45		90

**Table 6:** Treatment overview.

Treatment	Payment System	Framing	Subjects	N
CNS	**C**apitation	**N**eutral	**S**tudents	27
CMS	**C**apitation	**M**edical	**S**tudents	24
CMD	**C**apitation	**M**edical	**D**octors	12
FNS	**F**ee For Service	**N**eutral	**S**tudents	27
FMS	**F**ee For Service	**M**edical	**S**tudents	27
FMD	**F**ee For Service	**M**edical	**D**octors	9

**Table 7:** Payoff of subjects.

		*Patient* Payoff				*Physician* Payoff			*Insurer* Payoff		
Services Provided		L	M	H		FFS	CAP		L	M	H
1		58.5	4.5	4.5		15	50		85	85	40
2		85.5	31.5	4.5		30			85	85	40
3		58.5	58.5	4.5		45			85	85	40
4		31.5	85.5	31.5		60	50				40
5		4.5	58.5	58.5		75					40
6		4.5	31.5	85.5		90					40

**Notes:** Payoff of respective subject. Column 1 describes the number of Medical Services provided. Columns 2–4 present the expected *Patient* payoffs for the respective *Patient* type. Columns 5–6 show the *Physician* payoff in the Fee For Service and the Capitation treatments. Columns 7–9 show the *Insurer* payoff, dependent on the reported *Patient* type. Dots indicate non-achievable outcomes. The dashed line indicates the possible number of services provided into the amount achievable when the physician reports patient type L or M (1–3 units). When patient type H is reported, the budget is sufficient to provide up to six units of medical services.

**Table 8:** Deviation from optimal treatment between fee for service and capitation.

		Payment System		
***Patient***	**Fram.-Subj.**	**FFS**	**CAP**	**U-Test**
L	Neutr.-Stud.	**2.11**	0.04	***
	Med.-Stud.	**0.96**	0.08	***
	Med.-Doc.	0	0	
M	Neutr.-Stud.	**0.44**	**−1**	***
	Med.-Stud.	−0.04	**−0.83**	**
	Med.-Doc.	**−0.89**	**−1.08**	
H	Neutr.-Stud.	**−0.26**	**−0.63**	**
	Med.-Stud.	**−0.33**	**−0.54**	
	Med.-Doc.	0	**−0.75**	

**Notes:** Average provision of Medical Services across experimental conditions. Positive values indicate an overprovision of Medical Services. Negative values indicate underprovision of Medical Services. Bold formatted values are significantly different from zero (one-sided *t*-tests, *p* < 0.1). U-Test: Stars indicate *p*-values of Mann–Whitney U-tests of pairwise comparisons of misreporting/provided Medical Services between experimental conditions. * *p* < 0.1, ** *p* < 0.05, *** *p* <  0.01. Each subject decided about the reporting and provision of (medical) services of every *Patient* type. Therefore the total number of observations is 126. Number of observations in respective treatments: CNS—27, CMS—24, CMD—12, FNS—27, FMS—27, FMD—9.

**Table 9:** Deviation from optimal treatment between Neutral and Medical Framing.

		Framing		
***Patient***	**Payment System**	**Neutral**	**Medical**	**U-Test**
L	FFS	**2.11**	**0.96**	**
	CAP	0.04	0.08	
M	FFS	**0.44**	−0.04	
	CAP	**−1**	**−0.83**	
H	FFS	**−0.26**	**−0.33**	
	CAP	**−0.63**	**−0.54**	

**Notes:** Analysis only for student subject sample. Average provision of Medical Services across experimental conditions. Positive values indicate an overprovision of Medical Services. Negative values indicate underprovision of Medical Services. Bold formatted values are significantly different from zero (one-sided *t*-tests, *p* < 0.1). U-Test: Stars indicate *p*-values of Mann–Whitney U-tests of pairwise comparisons of provided Medical Services between experimental conditions. * *p* < 0.1, ** *p* < 0.05, *** *p* < 0.01. Each subject decided about the provision of (medical) services of every *Patient* type. Therefore, the total number of observations is 126. Number of observations in respective treatments: CNS—27, CMS—24, CMD—12, FNS—27, FMS—27, FMD—9.

**Table 10:** Deviation from optimal treatment between student and medical doctor samples.

		Subjects		
***Patient***	**Payment System**	**Students**	**Doctors**	**U-Test**
L	FFS	**0.96**	0	**
	CAP	0.08	0	
M	FFS	−0.04	**−0.89**	
	CAP	**−0.83**	**−1.08**	
H	FFS	**−0.33**	0	
	CAP	**−0.54**	**−0.75**	

**Notes:** Analysis only for treatments with medical framing. Average provision of Medical Services across experimental conditions. Positive values indicate an overprovision of Medical Services. Negative values indicate underprovision of Medical Services. Bold formatted values are significantly different from zero (one-sided *t*-tests, *p* < 0.1). U-Test: Stars indicate *p*-values of Mann–Whitney U-tests of pairwise comparisons of provided Medical Services between experimental conditions. * *p* < 0.1, ** *p* < 0.05, *** *p* < 0.01. Each subject decided about the reporting and provision of (medical) services of every *Patient* type. Therefore, the total number of observations is 126. Number of observations in respective treatments: CNS—27, CMS—24, CMD—12, FNS—27, FMS—27, FMD—9.

**Table 11:** Regression results—payoff for different participants by *patient* type.

		*Patient*	*Physician*	*Insurer*
*Patient* Type L	Fee For Service	−28.57 ***	−0.51	−10.51 ***
		(3.82)	(2.97)	(2.72)
	Medical Framing	11.02 ***	−8.76 ***	7.52 **
		(4.18)	(3.25)	(2.97)
	Medical Doctor	11.94 **	−5.68	4.23
		(5.56)	(4.32)	(3.96)
	Constant	74.79 ***	56.09 ***	77.76 ***
		(3.48)	(2.70)	(2.48)
*Patient* Type M	Fee For Service	−7.32 ***	10.46 ***	−15.27 ***
		(2.66)	(2.56)	(3.52)
	Medical Framing	5.72 **	−3.64	−3.96
		(2.91)	(2.80)	(3.85)
	Medical Doctor	−0.74	−5.37	9.30 *
		(3.88)	(3.73)	(5.12)
	Constant	56.66 ***	53.10 ***	77.64 ***
		(2.43)	(2.33)	(3.21)
*Patient* Type H	Fee For Service	8.66 **	36.30 ***	−2.85 *
		(4.16)	(1.65)	(1.69)
	Medical Framing	−0.31	−0.59	−0.65
		(4.55)	(1.80)	(1.85)
	Medical Doctor	1.93	2.27	0.09
		(6.06)	(2.40)	(2.46)
	Constant	71.17 ***	49.91 ***	43.93 ***
		(3.79)	(1.50)	(1.54)

**Notes:** Coefficients of seemingly unrelated regressions; Standard errors in parentheses; Number of observations in each estimation: 126. The table shows estimation results of three seemingly unrelated regressions, where each regression is either run with *Patients* of type L, M, or H; * p<0.1, ** p<0.05, *** p<0.01.

**Figure A3:**
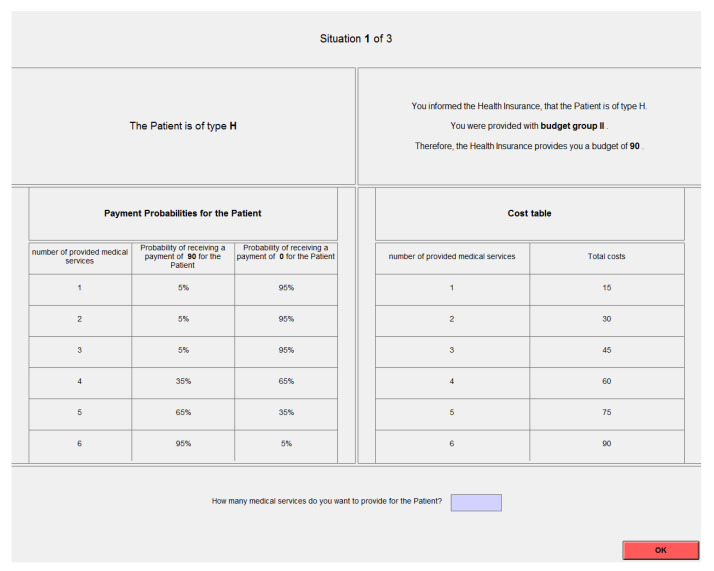
Provision stage.

## Appendix A. Reporting Behavior

Figure A1 shows the average deviation in reporting from the true type for each *Patient* type across the experimental conditions. The first row shows misreporting for the CAP treatments and misreporting in the FFS treatments can be found in the second row. In each row, the three columns indicate *Patient* type L, M, and H. In each of the six subfigures, average misreporting is indicated by the bars for students in the neutral framing, students in the medical framing, and doctors in the medical framing. Each bar also includes 95% confidence intervals. On average, we find that in our sample of students, *Patient* type is significantly overreported for type L. For type L and M *Patients*, misreporting is higher in the FFS treatments compared to the CAP treatments. There is no large difference between CAP and FFS for type H *Patients*. No clear pattern is visible with respect to framing or sample.

### Appendix A.1. Differences in Reporting between Fee for Service and Capitation

Table A1 compares the behavior in fee for service and capitation systems. For type L *Patients*, there are clear differences between physician payment systems: student subjects in the fee for service systems report significantly higher types than students in the capitation systems (CNS 0.22 vs. FNS 1 and CMS 0.13 vs. FMS 0.44). None of our medical doctor subjects misreports here.

*Patient* type is also overreported for type M *Patients*. Here, however, the difference between fee for service and capitation is only significantly different from zero in the neutrally framed experiments (CNS 0.04 vs. FNS 0.56). In the other two groups, physician payment system does not induce significantly different reporting behavior (CMS 0.33 vs. FMS 0.52 and CMD 0.08 vs. FMD 0.33. As for type M *Patients*, both under- and overreporting are possible, the average misreporting might deviate from the average absolute misreporting. However, Table A10 in the Appendix B shows that the average absolute misreporting is similar to the average misreporting.).

There is barely any deviation from true reporting for type H *Patients* with the exception of students in the neutral framing, who significantly underreport *Patient* type (CNS −0.15 vs. FNS 0). *Patient* and *Physician* payoff are aligned in the fee for service setting for type H *Patients*. However, this is not the case in the capitation setting, where participants face a trade-off between *Patient* and *Insurer* payoff, but not her own.

A reason for the observed significant underreporting of *Patient* type H in the neutrally framed capitation system could be that the participants are not made aware of the needs indicated by the medically framed *Patient*, and therefore value the payoff of the other participant more.

**Table A1:** Misreporting between fee for service and capitation.

		Payment System		
***Patient***	**Fram.-Subj.**	**FFS**	**CAP**	**U-Test**
	Neutr.-Stud.	**1**	**0.22**	***
L	Med.-Stud.	**0.44**	**0.13**	*
	Med.-Doc.	0	0	
	Neutr.-Stud.	**0.56**	0.04	***
M	Med.-Stud.	**0.52**	**0.33**	
	Med.-Doc.	**0.33**	0.08	
	Neutr.-Stud.	0	**−0.15**	*
H	Med.-Stud.	−0.04	−0.04	
	Med.-Doc.	0	−0.08	

**Notes:** Average misreporting across experimental conditions. Zero misreporting refers to the case where the true type equals the reported type. Bold formatted values are significantly different from zero (one-sided *t*-tests, *p* < 0.1). Columns 5 and 8: U-Test: Stars indicate *p*-values of Mann–Whitney U-tests of pairwise comparisons of misreporting/provided Medical Services between experimental conditions. * *p* < 0.1, ** *p* < 0.05, *** *p* < 0.01. Each subject decided on the reporting of every *Patient* type. Therefore, the total number of observations is 126. Number of observations in respective treatments: CNS—27, CMS—24, CMD—12, FNS—27, FMS—27, FMD—9.

### Appendix A.2. Differences in Reporting between Neutral and Medical Framing

To evaluate the influence of framing on reporting behavior, we compare the results of our student samples (Table A2). We find only small differences in the reporting behavior of students across neutrally and medically framed treatments. Misreporting is higher in the neutral framing for type L *Patients* (CNS 0.22 vs. CMS 0.13 and FNS 1 vs. FMS 0.44), but only the difference in the fee for service systems is statistically different from zero. The pattern is different for type M *Patients*. Here, overreporting is significantly higher in the medically framed capitation treatment (CNS 0.04 vs. CMS 0.33). Reporting for H type *Patients* does not differ between framings. For both framings we find significant average overreporting in the fee for service system. The magnitude was lower for *Patients* in the medically framed experiments, however the difference in overreporting between neutrally and medically framed treatments is only significant for *Patients* of type L.

**Table A2:** Misreporting between neutral and medical framing.

		Framing		
***Patient***	**Payment System**	**Neutral**	**Medical**	**U-Test**
L	FFS	**1**	**0.44**	**
	CAP	**0.22**	**0.13**	
M	FFS	**0.56**	**0.52**	
	CAP	0.04	**0.33**	**
H	FFS	0	−0.04	
	CAP	**−0.15**	−0.04	

**Notes:** Analysis only for student subject sample. Average misreporting across experimental conditions. Zero misreporting refers to the case where the true type equals the reported type. Bold formatted values are significantly different from zero (one-sided *t*-tests, *p* < 0.1). Columns 5 and 8: U-Test: Stars indicate *p*-values of Mann–Whitney U-tests of pairwise comparisons of misreporting Medical Services between experimental conditions. * *p* < 0.1, ** *p* < 0.05, *** *p* < 0.01. Each subject decided on the reporting of every *Patient* type. Therefore, the total number of observations is 126. Number of observations in respective treatments: CNS—27, CMS—24, CMD—12, FNS—27, FMS—27, FMD—9.

### Appendix A.3. Differences in Reporting between Student and Physician Samples

As a last comparison, we evaluate the effects of different subject pools by comparing the medically framed experiments of student and medical doctor subjects (Table A3). The reporting behavior of the students and medical doctors is very similar. The only significant difference is for type L *Patients* in the fee for service setting, where students on average overreport in contrast to the doctors, who report truthfully.

**Table A3:** Misreporting between student and medical doctor samples.

		Subjects		
***Patient***	**Payment System**	**Students**	**Doctors**	**U-Test**
L	FFS	**0.44**	0	*
	CAP	**0.13**	0	
M	FFS	**0.52**	**0.33**	
	CAP	**0.33**	0.08	
H	FFS	−0.04	0	
	CAP	−0.04	−0.08	

**Notes:** Analysis only for treatments with medical framing. Average misreporting across experimental conditions. Zero misreporting refers to the case where the true type equals the reported type. Bold formatted values are significantly different from zero (one-sided *t*-tests, *p* < 0.1). U-Test: Stars indicate *p*-values of Mann–Whitney U-tests of pairwise comparisons of misreporting between experimental conditions. * *p* < 0.1, ** *p* < 0.05, *** *p* < 0.01. Each subject decided on the reporting of every *Patient* type. Therefore, the total number of observations is 126. Number of observations in respective treatments: CNS—27, CMS—24, CMD—12, FNS—27, FMS—27, FMD—9.

### Appendix A.4. Provision Conditional on Reporting

In order to gain insights into the motivation behind our subjects’ behavior, we analyze the Medical Service provision conditional on reporting for the different *Patient* types. A detailed overview on our participants’ behavior is given in Table A12, Table A13 and Table A14 (in the Appendix B).

For type L *Patients*, misreporting is not necessary to obtain a budget that is sufficient for optimal Medical Service provision. Therefore, misreporting cannot be explained by *Patient*-oriented motives. In the fee for service setting, the *Physician* has an incentive to overreport, as the resulting budget enables him/her to provide a higher number of services, which increases his/her personal payoff. More than half of the participants in the neutrally framed fee for service setting overreport, where the vast majority then provide the maximum number of services in order to maximize their own profit.

For type M *Patients*, overreporting is necessary to obtain a budget which is sufficient for optimal Medical Service provision. In the capitation setting, overreporting can only be motivated by external factors i.e., providing the optimal number of services for the *Patient* (or harming the *Insurer*). In the majority of cases, overreporting is used to provide the optimal number of services for the *Patient*. In the fee for service settings with *Patients* of type M, overreporting can be motivated by the personal financial incentive, the willingness to provide the optimal number of services for the *Patient*, or a combination of both. Providing the maximum number of services (overprovision by two) is motivated fully by personal interests, while overproviding by one could partly be motivated by earning more personally but also not harming the *Patient* more than he/she would have been harmed when the doctor reported his/her true type to the *Insurer*. The neutral framing mainly leads to *Physicians* maximizing their own payoff by providing the maximum number of services. Although the majority of participants also provides the maximum number of services in the medically framed setting, a larger portion either chooses the optimal number of services or only partially overprovides.

The medical doctors in our sample use the overreporting not to maximize their own payoff, but to treat the *Patients* optimally (One medical doctor underprovides even though she overreports for the type M *Patient*.). Observed differences between our neutrally framed and the medically framed experiments suggest that the medical framing induces more *Patient*-oriented behavior, whereas the neutral framing leads to more self-centered, individual payoff maximizing behavior.

## Appendix B. Additional Tables

**Table A4:** Misreporting of Type L *Patients* across experimental conditions.

Treatment	Avg. Misreporting		Treatment	CNS	CMS	CMD	FNS	FMS
CNS	0.22 **		CNS					
CMS	0.13 *		CMS					
CMD	0		CMD					
FNS	1 ***		FNS	***	***	***		
FMS	0.44 ***		FMS		*	*	**	
FMD	0		FMD				***	*

**Notes:***Left table*: Average misreporting across treatments. Zero misreporting refers to the case where the True Type (L) equals the Reported Type (L). Overreporting by one/two refers to the case where M/H is reported, whereas the True Type is L. Stars indicate *p*-values of one-sided *t*-tests, testing whether there is statistically significant overreporting. *Right table*: Stars indicate *p*-values of Mann–Whitney U-tests of pairwise comparisons of (mis)reporting between treatments. * *p* < 0.1, ** *p* < 0.05, *** *p* < 0.01. No star means no significant difference at *p* < 0.1. Dots indicate comparisons already represented by other combinations of rows and columns.

**Table A5:** Misreporting of Type M *Patients* across experimental conditions.

Treatment	Avg. Misreporting		Treatment	CNS	CMS	CMD	FNS	FMS
CNS	0.04		CNS					
CMS	0.33 ***		CMS	**				
CMD	0.08		CMD					
FNS	0.56 ***		FNS	***		***		
FMS	0.52 ***		FMS	***		**		
FMD	0.33 **		FMD	*				

**Notes:***Left table*: Average misreporting across treatments. Zero misreporting refers to the case where the True Type (M) equals the Reported Type (M). Overreporting/underreporting by +1/−1 refers to the case where H/L is reported, whereas the True Type is M. Stars indicate p-values of one-sided t-tests, testing whether there is statistically significant overreporting/underreporting. *right table*: Stars indicate p-values of Mann–Whitney U-tests of pairwise comparisons of (mis)reporting between treatments. * *p*  < 0.1, ** *p* < 0.05, *** *p* < 0.01. No star means no significant difference at *p* < 0.1. Dots indicate comparisons already represented by other combinations of rows and columns.

**Table A6:** Misreporting of Type H *Patients* across Experimental Conditions.

Treatment	Avg. Misreporting		Treatment	CNS	CMS	CMD	FNS	FMS
CNS	−0.15 *		CNS					
CMS	−0.04		CMS					
CMD	−0.08		CMD					
FNS	0		FNS	*				
FMS	−0.04		FMS					
FMD	0		FMD					

**Notes:***Left table*: Average misreporting across treatments. Zero misreporting refers to the case where the True Type (H) equals the Reported Type (H). Underreporting by one/two refers to the case where M/L is reported, whereas the True Type is H. Stars indicate *p*-values of one-sided *t*-tests, testing whether there is statistically significant underreporting. *Right table*: Stars indicate *p*-values of Mann–Whitney U-tests of pairwise comparisons of (mis)reporting between treatments. * *p* < 0.1, ** *p* < 0.05, *** *p* < 0.01. No star means no significant difference at *p* < 0.1. Dots indicate comparisons already represented by other combinations of rows and columns.

**Table A7:** Deviation from optimal treatment of Type L *Patients* across experimental conditions.

Treatment	Avg. Misreporting		Treatment	CNS	CMS	CMD	FNS	FMS
CNS	0.04		CNS					
CMS	0.08		CMS					
CMD	0		CMD					
FNS	2.11 ***		FNS	***	***	***		
FMS	0.96 ***		FMS	***	***	***	**	
FMD	0		FMD				***	**

**Notes:***Left table*: Average provision of (medical) services across treatments. Positive values indicate an overprovision of (medical) Services. Negative values indicate underprovision of (medical) services. Stars indicate *p*-values of one-sided *t*-tests, testing whether the mean provision of (medical) services differs significantly from 0 (optimal number of provided Services). *Right table*: Stars indicate *p*-values of Mann–Whitney U-tests of pairwise comparisons of provided (medical) services between treatments. * *p* < 0.1, ** *p* < 0.05, *** *p* < 0.01. No star means no significant difference at *p* < 0.1. Dots indicate comparisons already represented by other combinations of rows and columns.

**Table A8:** Deviation from optimal treatment of Type M *Patients* across experimental conditions.

Treatment	Avg. Maltreatment		Treatment	CNS	CMS	CMD	FNS	FMS
CNS	−1 ***		CNS					
CMS	−0.83 ***		CMS					
CMD	−1.08 ***		CMD					
FNS	0.44 *		FNS	***	***	***		
FMS	−0.04		FMS	***	**	**		
FMD	−0.89 ***		FMD				**	

**Notes:***Left table*: Average provision of (medical) services across treatments. Positive values indicate an overprovision of (medical) Services. Negative values indicate underprovision of (medical) services. Stars indicate *p*-values of one-sided *t*-tests, testing whether the mean provision of (medical) services differs significantly from 0 (optimal number of provided Services). *Right table*: Stars indicate *p*-values of Mann–Whitney U-tests of pairwise comparisons of provided (medical) services between treatments. * *p* < 0.1, ** *p* < 0.05, *** *p* < 0.01. No star means no significant difference at *p* < 0.1. Dots indicate comparisons already represented by other combinations of rows and columns.

**Table A9:** Deviation from optimal treatment of Type H *Patients* across experimental conditions.

Treatment	Avg. Maltreatment Treatment	CNS	CMS	CMD	FNS	FMS
CNS	−0.63 ***		CNS					
CMS	−0.54 **		CMS					
CMD	−0.75 *		CMD					
FNS	−0.26 *		FNS	**				
FMS	−0.33 **		FMS					
FMD	0		FMD	*				

**Notes:***Left table*: Average provision of (medical) services across treatments. Positive values indicate an overprovision of (medical) services. Negative values indicate underprovision of (medical) services. Stars indicate *p*-values of one-sided *t*-tests, testing whether the mean Provision of (medical) services differs significantly from 0 (optimal number of provided services). *Right table*: Stars indicate *p*-values of Mann–Whitney U-tests of pairwise comparisons of provided (medical) services between treatments. * *p* < 0.1, ** *p* < 0.05, *** *p* < 0.01. No star means no significant difference at *p* < 0.1. Dots indicate comparisons already represented by other combinations of rows and columns.

**Table A10:** Absolut Misreporting of Type M *Patients* across Experimental Conditions.

Treatment	Avg. Maltreatment		Treatment	CNS	CMS	CMD	FNS	FMS
CNS	0.11 **		CNS					
CMS	0.33 ***		CMS	*				
CMD	0.08		CMD					
FNS	0.63 ***		FNS	***	**	***		
FMS	0.52 ***		FMS	***		**		
FMD	0.33 **		FMD					

**Notes:***Left table*: Average absolute misreporting across treatments. Zero misreporting refers to the case where the True Type (M) equals the Reported Type (M). Stars indicate *p*-values of one-sided *t*-tests, testing whether there is statistically significant misreporting. *Right table*: Stars indicate *p*-values of Mann–Whitney U-tests of pairwise comparisons of misreporting between treatments. * *p* < 0.1, ** *p* < 0.05, *** *p* < 0.01. No star means no significant difference at *p* < 0.1. Dots indicate comparisons already represented by other combinations of rows and columns.

**Table A11:** Absolute deviation from optimal treatment of Type M *Patients* across experimental conditions.

Treatment	Avg. Maltreatment		Treatment	CNS	CMS	CMD	FNS	FMS
CNS	1 ***		CNS					
CMS	0.83 ***		CMS					
CMD	1.08 ***		CMD					
FNS	1.41 ***		FNS	***	***			
FMS	1.15 ***		FMS		*			
FMD	0.89 ***		FMD				*	

**Notes:***Left table*: Average absolute deviation from optimal treatment across treatments. Stars indicate *p*-values of one-sided *t*-tests, testing whether the mean absolute provision of medical services differs significantly from 0 (optimal number of provided services). *Right table*: Stars indicate *p*-values of Mann–Whitney U-tests of pairwise comparisons of provided medical services between treatments. * *p* < 0.1, ** *p* < 0.05, *** *p* < 0.01. No star means no significant difference at *p* < 0.1. Dots indicate comparisons already represented by other combinations of rows and columns.

**Table A12:** Reporting and provision of medical services for Type L *Patients*.

Treatment	Reported Type	Provided Services						
		1	2	3	4	5	6	Obs.
	L	0	**23**	0				23
CNS	M	0	**1**	1				2
	H	1	**0**	1	0	0	0	2
	L	0	**22**	0				22
CMS	M	0	**1**	0				1
	H	0	**0**	0	1	0	0	1
	L	0	**12**	0				12
CMD	M	0	**0**	0				0
	H	0	**0**	0	0	0	0	0
		**1**	**2**	**3**	**4**	**5**	**6**	**Obs.**
	L	1	**4**	8				13
FNS	M	0	**0**	1				1
	H	0	**0**	1	0	0	12	13
	L	2	**8**	10				20
FMS	M	0	**0**	2				2
	H	0	**1**	0	0	0	4	5
	L	0	**9**	0				9
FMD	M	0	**0**	0				0
	H	0	**0**	0	0	0	0	0

**Notes:** Dots indicate non-achievable outcomes. Bold formatted values represent the optimal number of medical services for the *Patient* of type L.

**Table A13:** Reporting and provision of medical services for Type M *Patients*.

Treatment	Reported Type	Provided Services						
		1	2	3	4	5	6	Obs.
	L	0	0	1				1
CNS	M	0	1	23				24
	H	0	0	1	**1**	0	0	2
	L	0	0	0				0
CMS	M	0	0	16				16
	H	0	2	0	**6**	0	0	8
	L	0	0	0				0
CMD	M	0	2	9				11
	H	0	0	0	**1**	0	0	1
	L	1	0	0				1
FNS	M	0	0	10				10
	H	0	0	0	**2**	3	11	16
	L	0	0	0				0
FMS	M	0	2	11				13
	H	0	0	1	**3**	5	5	14
	L	0	0	0				0
FMD	M	0	1	5				6
	H	0	0	1	**2**	0	0	3

**Notes:** Dots indicate non-achievable outcomes. Bold formatted values represent the optimal number of medical services for the *Patient* of type M.

**Table A14:** Reporting and provision of medical services for Type H *Patients*.

Treatment	Reported Type	Provided Services						
		1	2	3	4	5	6	Obs.
	L	1	0	0				1
CNS	M	0	0	2				2
	H	0	0	0	1	4	**19**	24
	L	0	0	0				0
CMS	M	0	0	1				1
	H	1	0	1	1	0	**20**	23
	L	0	0	0				0
CMD	M	1	0	0				1
	H	0	0	1	0	1	**9**	11
	L	0	0	0				0
FNS	M	0	0	0				0
	H	1	0	0	1	0	**25**	27
		**1**	**2**	**3**	**4**	**5**	**6**	**Obs.**
	L	0	0	0				0
FMS	M	0	1	0				1
	H	0	0	0	1	3	**22**	26
	L	0	0	0				0
FMD	M	0	0	0				0
	H	0	0	0	0	0	**9**	9

**Notes:** Dots indicate non-achievable outcomes. Bold formatted values represent the optimal number of medical services for the *Patient* of type H.

**Table A15:** Regression results—payoff for different participants by *Patient* Type.

		*Patient*			*Physician*			Insurer	
*Patient* Type L	Fee For Service	−28.57 ***	−29.06 ***		−0.51	−0.46		−10.51 ***	−10.91 ***
		(3.82)	(3.83)		(2.97)	(2.96)		(2.72)	(2.72)
	Medical Framing	11.02 ***	10.75 **		−8.76 ***	−9.29 ***		7.52 **	7.28 **
		(4.18)	(4.31)		(3.25)	(3.33)		(2.97)	(3.06)
	Medical Doctor	11.94 **	9.50		−5.68	−4.35		4.23	6.18
		(5.56)	(9.26)		(4.32)	(7.15)		(3.96)	(6.57)
	Age	-	0.25		-	−0.16		-	−0.08
		-	(0.52)		-	(0.40)		-	(0.37)
	Female	-	6.92		-	−7.04 **		-	4.73
		-	(4.27)		-	(3.29)		-	(3.03)
	Pro Social	-	−4.24		-	1.75		-	−4.11
		-	(4.02)		-	(3.10)		-	(2.85)
	Risk	-	0.43		-	−0.24		-	0.41
		-	(0.89)		-	(0.69)		-	(0.63)
	Constant	74.79 ***	64.98 ***		56.09 ***	64.59 ***		77.76 ***	76.79 ***
		(3.48)	(12.90)		(2.70)	(9.96)		(2.48)	(9.15)
*Patient* Type M	Fee For Service	−7.32 ***	−7.55 ***		10.46 ***	11.12 ***		−15.27 ***	−15.80 ***
		(2.66)	(2.60)		(2.56)	(2.51)		(3.52)	(3.50)
	Medical Framing	5.72 **	5.83 **		−3.64	−2.90		−3.96	−4.72
		(2.91)	(2.93)		(2.80)	(2.82)		(3.85)	(3.94)
	Medical Doctor	−0.74	11.15 *		−5.37	5.36		9.30 *	−4.33
		(3.88)	(6.29)		(3.73)	(6.06)		(5.12)	(8.47)
	Age	-	−0.77 **		-	−0.81 **		-	0.99 **
		-	(0.35)		-	(0.34)		-	(0.47)
	Female	-	3.98		-	−5.30 *		-	3.76
		-	(2.90)		-	(2.79)		-	(3.90)
	Pro Social	-	−5.29 *		-	2.43		-	−0.96
		-	(2.73)		-	(2.63)		-	(3.67)
	Risk	-	0.49		-	−0.40		-	0.12
		-	(0.60)		-	(0.58)		-	(0.81)
	Constant	56.66 ***	72.18 ***		53.10 ***	75.29 ***		77.64 ***	52.82 ***
		(2.43)	(8.77)		(2.33)	(8.44)		(3.21)	(11.80)
*Patient* Type H	Fee For Service	8.66 **	6.84 *		36.30 ***	36.03 ***		−2.85 *	−2.71
		(4.16)	(4.09)		(1.65)	(1.65)		(1.69)	(1.71)
	Medical Framing	−0.31	−3.97		−0.59	−1.05		−0.65	−0.14
		(4.55)	(4.61)		(1.80)	(1.86)		(1.85)	(1.92)
	Medical Doctor	1.93	2.06		2.27	5.91		0.09	3.95
		(6.06)	(9.88)		(2.40)	(3.99)		(2.46)	(4.13)
	Age	-	0.02		-	−0.25		-	−0.26
		-	(0.55)		-	(0.22)		-	(0.23)
	Female	-	−3.21		-	−0.90		-	0.93
		-	(4.55)		-	(1.84)		-	(1.90)
	Pro Social	-	−11.80 ***		-	−2.07		-	0.48
		-	(4.29)		-	(1.73)		-	(1.79)
	Risk	-	0.60		-	0.22		-	0.17
		-	(0.95)		-	(0.38)		-	(0.40)
	Constant	71.17 ***	77.16 ***		49.91 ***	56.37 ***		43.93 ***	48.13 ***
		(3.79)	(13.77)		(1.50)	(5.57)		(1.54)	(5.75)

**Notes**: Coefficients of seemingly unrelated regressions; Standard errors in parentheses; Number of observations in each estimation: 126. The table shows estimation results of three seemingly unrelated regressions, where each regression is either run with *Patient* of type L, M, or H; * *p* < 0.1, ** *p* < 0.05, *** *p* < 0.01. Description of additional variables: “Pro Social” (subjects with a cooperative/pro social attitude obtained from social value orientation slider measure [52], the reference category are subjects with individualist preferences), “Risk” (subjects where asked the following question: “Are you generally willing to take risks or are you trying to avoid risks?” Possible answers ranged from zero to ten, where zero represents “not willing to take risks” and ten represents “very willing to take risks”).

## Appendix C. Instructions Neutral Framing

Capitation/Fee For Service

### General Information

Welcome! Today you participate in an economic experiment.

You receive 4 Euro for showing up on time. In the course of the experiment you can earn additional money. Therefore, please take the time to read the following instructions carefully.

You will make your decisions individually and anonymously at your place. During the experiment please do not communicate with the other participants and do not use your mobile phone. If you violate these rules we will exclude you from the experiment without any payment.

If you have any questions please raise your hand. We will then come to your place and answer the question. In the experiment we will use the currency “Taler”.

The payment for each participant will be converted into Euros at the end of the experiment and paid out in cash. The exchange rate is **10 Taler = 1 Euro**. The payment will be anonymous, i.e., no other participant will be informed about your payment.

### The Experiment

**Group formation and types of participants**

**Groups with three persons** will be randomly formed before the beginning of the experiment. This group composition will not change during the whole experiment—you will always stay in the group with the same two people. You will have nothing to do with the other groups and their members during the whole experiment.

Within the groups there will be each one of three types of participants: **A**, **B**, and **C**. The roles within one group are randomly assigned.

Only participant B can influence the payments of his/her group members with his/her decisions. Participant A and participant C will not make any decisions in the experiment.

**Course of the experiment**

Until the end of the experiment no one of the three group members knows which participant role has been assigned to him/her. At first every group member makes decisions, as if he/she was in the role of participant B. After all group members made their decision in the role of participant B, it will be announced how the roles inside the group have been randomly assigned. Only the decisions of that group member who was assigned the role of participant B are relevant for the payment and influence the payments of the group members. The decisions of those group members who are assigned roles of participant A or C are not relevant for the payment.

**Relationship between the participants**

Participant A needs services from participant B. The payment of participant A is influenced by the number of services that he/she receives from participant B. The services are associated with costs. In order to cover costs, participant B needs a budget that he/she has to request from participant C. While requesting the budget, participant B reports information about participant A to participant C. The number of the budget depends on which information about participant A has been reported to participant C via participant B. After participant B received the budget, which was subtracted from the endowment of participant C, he/she decides which number of service he/she wants to provide for participant A. The following picture illustrates the relationship between the participants of the experiment.

**Description of the participants and their payments**

**Participant A**

Participant A can take on one of **three possible types: Type L, M, or H**. He/she does not learn about their own type and does not make own decisions. His/her possible payment depends on his/her type and on the number of services that participant B provides.

**The payment of participant A can take on two possible values: 90 Taler or 0 Taler. The number of provided services determines the probability of occurrence of these possible payments.**

Depending on the type of participant A the probability of a payment of 90 is maximized by different numbers of services:

Type	Optimal number of services
L	2 units
M	4 units
H	6 units

A payment of 90 Taler is more probable the closer the number of provided services is to the optimal number of services for participant A:

- If the number of services provided by participant B is optimal for participant A, he/she receives a payment of 90 Taler with a probability of 95%. With a probability of 5% she receives a payment of 0 Taler.
- If the number of actually provided services by participant B deviates by one unit from the optimal number of services for participant A, participant A receives a payment of 90 Taler with a probability of 65%. With a probability of 35% he/she receives a payment of 0 Taler.
- If the number of actually provided services by participant B deviates by two units from the optimal number of services for participant A, participant A receives a payment of 90 Taler with a probability of 35%. With a probability of 65% he/she receives a payment of 0 Taler.
- If the number of actually provided services by participant B deviates by three units or more from the optimal number of services for participant A, participant A receives a payment of 90 Taler with a probability of 5%. With a probability of 95% he/she receives a payment of 0 Taler.

The following three tables provide an overview of all payments of the tree possible types of participant A, depending on the number of services provided by participant B.

	Participant A of **type L**	
**Number of services** provided	Probability for	Probability for
by participant B	payment of **90**	payment of **0**
1	65%	35%
2	95%	5%
3	65%	35%
4	35%	65%
5	5%	95%
6	5%	95%

	Participant A of **type M**	
**Number of services** provided	Probability for	Probability for
by participant B	payment of **90**	payment of **0**
1	5%	95%
2	35%	65%
3	65%	35%
4	95%	5%
5	65%	35%
6	35%	65%

	Participant A of **type H**	
**Number of services** provided	Probability for	Probability for
by participant B	payment of **90**	payment of **0**
1	5%	95%
2	5%	95%
3	5%	95%
4	35%	35%
5	65%	65%
6	95%	5%

The group member which has been assigned to the role of participant A learns at the end of the experiment how high their payment is.

**Participant B**

Participant B is confronted with the different types of participant A in three situations occurring in random order and has to make decisions. After he/she made a decision in all the situations, **one** situation will be randomly selected. The decisions made in this selected situation determine the payments of the group members. Each situation in this experiment will be given the relevant payment equally often, i.e., all situations are equally likely.

**Only participant B learns about the type of participant A. Neither participant A nor participant C will learn the type of participant A at any time.**

Participant B decides in every situation which number of services he/she wants to provide for participant A.

**The payment of participant B is independent of the number of services that he/she provides for participant A. Participant B receives 50 Taler in every situation.**

**The payment of participant B is dependent on the number of services that he/she provides for participant A. Participant B receives 15 Taler per unit of service provided.**

The provided services are associated with **costs**. Every unit of service provided costs 15 Taler. These costs are not incurred by participant B but are financed by a **budget**, which is subtracted from the endowment of participant C and has to be requested by participant B. Therefore, participant B informs participant C about participant A’s type. If participant B informs participant C that participant A is a type L or M, he/she will be automatically provided the budget group I (45 Taler). If he/she reports that participant A is type H, then he/she will be automatically provided with budget group II (90 Taler). The budget available is automatically subtracted from the endowment of participant C.

Participant B cannot exceed the budget available.

The group member that has been assigned to the role of participant B, learns at the end of the experiment which situation is payment relevant. He/she also learns which payments resulted from his/her decisions for participant A and participant C.

**Participant C**

Participant C does not learn which type participant A is and does not make any own decisions. Participant C owns an **endowment of 130 Taler**. The information about participant A reported by participant B determines automatically the provided budget. Participant C cannot influence the size of the budget available.

**The available budget is subtracted from the endowment of participant C. The remaining endowment determines the payment for participant C.**

The group member who is assigned to the role of participant C, learns at the end of the experiment which information he/she got from participant B in the randomly assigned situation and which number of services participant B provided for participant A in this situation.

The following two tables provide an overview of the budget groups and the costs.

**Budget Group**				**Cost Table**	
**Type**	**Budged Group**	**Budget**		**Service Units**	**Total Costs**
L	I	45		1	15
				2	30
M				3	45
H	II	90		4	60
				5	75
				6	90

The budget, which is not used by participant B for provision of services, does not benefit any of the participants A, B, or C.

**Summary of the course of a situation**

(1) Participant B learns in every situation which of the three possible types participant A is in the current case. Participant A and participant C do not have any information about the type of participant A at any point of time.
(2) Participant B tells participant C which type participant A is.
(3) On the basis of his/her message about participant A, participant B will be provided a budget group. The budget associated with that will be subtracted from the endowment of participant C.
(4) Participant B decides which number of services she wants to provide for participant A.

**Summary of payment determination**

At the end of the experiment it is announced how the roles for participants A, B, and C have been randomly assigned within each group. Only the decisions of that group member which has been assigned to the role of participant B are payment relevant and influence the payments of the group members. Afterwards, one of the three situations is randomly chosen. The payments for each of the participants result from the decision of participant B in this situation as follows.

**Payment of participant A**

The payment of participant A is determined by the number of services provided by participant B. The closer the provided number of services is to the optimal number of services provided, the higher the likelihood that participant A receives a payment of 90 Taler. The further the provided number of services deviates from the optimal number of services provided, the higher the likelihood that participant A receives a payment of 0 Taler.

**Payment of participant B**

The payment of participant B is independent from the number of services provided for participant A. Participant B receives 50 Taler in every situation. They payment of participant B is dependent on the number of service provided for participant A. Participant B receives 15 Taler per unit of service provided.

**Payment of participant C**

The endowment of participant C is 130 Taler. The budget available connected to the requested budget group is subtracted from the endowment of participant C. The remaining endowment determines the payment of participant C.

**You reached the end of the instructions. You can continue by clicking OK on the screen.**

## Appendix D. Instructions Medical Framing

Capitation/Fee For Service

### General Information

Welcome! Today you participate in an economic experiment.

You receive 4 Euro for showing up on time. In the course of the experiment you can earn additional money. Therefore, please take the time to read the following instructions carefully.

You will make your decisions individually and anonymously at your place. During the experiment please do not communicate with the other participants and do not use your mobile phone. If you violate these rules we will exclude you from the experiment without any payment.

If you have any questions please raise your hand. We will then come to your place and answer the question. In the experiment we will use the currency “Taler”.

The payment for each participant will be converted into Euros at the end of the experiment and paid out in cash. The exchange rate is **10 Taler = 1 Euro**. The payment will be anonymous, i.e., no other participant will be informed about your payment.

### The Experiment

**Group formation and types of participants**

**Groups with three persons** will be randomly formed before the beginning of the experiment. This group composition will not change during the whole experiment—you will always stay in the group with the same two people. You will have nothing to do with the other groups and their members during the whole experiment.

Within the groups there will be each one of three types of participants: **Patient, Physician, and Health Insurance**. The roles within one group are randomly assigned.

Only the Physician can influence the payments of his/her group members with his/her decisions. The Patient and Health Insurance roles will not make any decisions in the experiment.

**Course of the experiment**

Until the end of the experiment no one of the three group members knows which participant role has been assigned to him/her. At first every group member makes decisions, as if he/she was in the role of the Physician. After all group members made their decision in the role of the Physician, it will be announced how the roles inside the group have been randomly assigned. Only the decisions of that group member who was assigned the role of the Physician are relevant for the payment and influence the payments of the group members. The decisions of those group members who are assigned roles of Patient or Health Insurance are not relevant for the payment.

**Relationship between the participants**

The Patient needs Medical Services from the Physician. The payment of the Patient is influenced by the number of Medical Services that he/she receives from the Physician. The Medical Services are associated with costs. In order to cover costs, the Physician needs a budget that he/she has to request from the Health Insurance. While requesting the budget, the Physician reports information about the Patient to the Health Insurance. The number of the budget depends on which information about the Patient has been reported to the Health Insurance via the Physician. After the Physician received the budget, which was subtracted from the endowment of the Health Insurance, he/she decides which number of Medical Service he/she wants to provide for the Patient. The following picture illustrates the relationship between the participants of the experiment.

**Description of the participants and their payments**

**Patient**

The Patient can take on one of **three possible types: Type L, M, or H**. He himself/she herself does not learn about her own type and does not make own decisions. His/her possible payment depends on his/her type and on the number of Medical Services that the Physician provides.

**The payment of the Patient can take on two possible values: 90 Taler or 0 Taler. The number of provided Medical Services determines the probability of occurrence of these possible payments.**

Depending on the type of the Patient, the probability of a payment of 90 is maximized by different numbers of Medical Services:

Type	Optimal number of Medical Services
L	2 units
M	4 units
H	6 units

A payment of 90 Taler is more probable the closer the number of provided Medical Services is to the optimal number of Medical Services for participant A:

- If the number of Medical Services provided by the Physician is optimal for the Patient, he/she receives a payment of 90 Taler with a probability of 95%. With a probability of 5% she receives a payment of 0 Taler.
- If the number of actually provided Medical Services by the Physician deviates by one unit from the optimal number of Medical Services for the Patient, the Patient receives a payment of 90 Taler with a probability of 65%. With a probability of 35% he/she receives a payment of 0 Taler.
- If the number of actually provided Medical Services by the Physician deviates by two units from the optimal number of Medical Services for the Patient, the Patient receives a payment of 90 Taler with a probability of 35%. With a probability of 65% he/she receives a payment of 0 Taler.
- If the number of actually provided Medical Services by the Physician deviates by three units or more from the optimal number of Medical Services for the Patient, the Patient receives a payment of 90 Taler with a probability of 5%. With a probability of 95% he/she receives a payment of 0 Taler.

The following three tables provide an overview of all payments of the tree possible types of the Patient, depending on the number of Medical Services provided by the Physician.

	Patient of **type L**	
**Number of Medical Services** provided	Probability for	Probability for
by participant B	payment of **90**	payment of **0**
1	65%	35%
2	95%	5%
3	65%	35%
4	35%	65%
5	5%	95%
6	5%	95%

	Patient of **type M**	
**Number of Medical Services** provided	Probability for	Probability for
by participant B	payment of **90**	payment of **0**
1	5%	95%
2	35%	65%
3	65%	35%
4	95%	5%
5	65%	35%
6	35%	65%

	Patient of **type H**	
**Number of Medical Services** provided	Probability for	Probability for
by participant B	payment of **90**	payment of **0**
1	5%	95%
2	5%	95%
3	5%	95%
4	35%	35%
5	65%	65%
6	95%	5%

The group member which has been assigned to the role of the Patient learns at the end of the experiment how high their payment is.

**Physician**

The Physician is confronted with the different types of the Patient in three situations occurring in random order and has to make decisions. After he/she made a decision in all the situations, **one** situation will be randomly selected. The decisions made in this selected situation determine the payments of the group members. Each situation in this experiment will be payment relevant equally often, i.e., all situations are equally likely.

**Only the Physician learns about the type of the Patient. Neither the Patient nor the Health Insurance will learn the type of the Patient at any time.**

The Physician decides in every situation which number of Medical Services he/she wants to provide for the Patient.

**The payment of the Physician is independent of the number of Medical Services that he/she provides for the Patient. The Physician receives 50 Taler in every situation.**

**The payment of the Physician is dependent on the number of Medical Services that he/she provides for the Patient. The Physician receives 15 Taler per unit of Medical Services provided.**

The provided Medical Services are associated with **costs**. Every unit of Medical Services provided costs 15 Taler. These costs are not incurred by the Physician but are financed by a **budget**, which is subtracted from the endowment of the Health Insurance and has to be requested by the Physician. Therefore, the Physician informs the Health Insurance about the Patient’s type. If the Physician informs the Health Insurance that the Patient is a type L or M, he/she will be automatically provided the budget group I (45 Taler). If he/she reports that the Patient is type H, then she will be automatically provided with budget group II (90 Taler). The budget available is automatically subtracted from the endowment of the Health Insurance.

Participant B cannot exceed the budget available.

The group member that has been assigned to the role of the Physician learns at the end of the experiment which situation is payment relevant. He/she also learns which payments resulted from his/her decisions for the Patient and the Health Insurance.

**Health Insurance**

The Health Insurance does not learn which type the Patient is and does not make any of its own decisions. The Health Insurance owns an **endowment of 130 Taler**. The information about the Patient reported by the Physician automatically determines the provided budget. The Health Insurance cannot influence the size of the budget available.

**The available budget is subtracted from the endowment of the Health Insurance. The remaining endowment determines the payment for the Health Insurance.**

The group member who is assigned to the role of the Health Insurance learns at the end of the experiment which information he/she received from the Physician in the randomly assigned situation and which number of Medical Services the Physician provided for the Patient in this situation.

The following two tables provide an overview of the budget groups and the costs.

**Budget Group**				**Cost Table**	
**Type**	**Budged Group**	**Budget**		**Service Units**	Total Costs
L	I	45		1	15
				2	30
M				3	45
H	II	90		4	60
				5	75
				6	90

Budget, which is not used by participant B for the provision of Medical Services, does not benefit the Patient, the Physician, or the Health Insurance.

**Summary of the course of a situation**

(1) The Physician learns in every situation which of the three possible types the Patient is in the current case. The Patient and the Health Insurance do not have any information about the type of the Patient at any point of time.
(2) The Physician tells the Health Insurance which type the Patient is.
(3) On the basis of her message about the Patient, the Physician will be provided a budget group. The budget associated with that will be subtracted from the endowment of the Health Insurance.
(4) The Physician decides which number of Medical Services she wants to provide for the Patient.

**Summary of payment determination**

At the end of the experiment it is announced how the roles for Patient, Physician, and Health Insurance have been randomly assigned within each group. Only the decisions of that group member which has been assigned to the role of the Physician are payment relevant and influence the payments of the group members. Afterwards, one of the three situations is randomly chosen. The payments for each of the participants result from the decision of the Physician in this situation as follows.

**Payment of Patient**

The payment of the Payment is determined by the number of Medical Services provided by the Physician. The closer the provided number of Medical Services is to the optimal number of Medical Services provided, the higher is the likelihood that the Patient receives a payment of 90 Taler. The further the provided number of Medical Services deviates from the optimal number of Medical Services provided, the higher is the likelihood that the Patient receives a payment of 0 Taler.

**Payment of Physician**

The payment of the Physician is independent from the number of Medical Services provided for the Patient. The Physician receives 50 Taler in every situation. They payment of the Physician is dependent on the number of Medical Services provided for the Patient. The Physician receives 15 Taler per unit of Medical Services provided.

**Payment of Health Insurance**

The endowment of the Health Insurance is 130 Taler. The budget available connected to the requested budget group is subtracted from the endowment of the Health Insurance. The remaining endowment determines the payment of the Health Insurance.

**You reached the end of the instructions. You can continue by clicking OK on the screen.**

## Appendix E. Control Questions Neutral Framing

The subjects had to answer questions among two blocks of answer categories. In the first block, the possible answers were either “*right*” or “*wrong*”. If a subject clicked on “*wrong*”, although “*right*” would have been correct, they were informed: “*Your answer is not correct. Please change your entry.*” This information also appeared if a subject provided a wrong answer in the second question block of open questions. Subjects were asked to raise their hand, whenever they had a question. At the beginning of the control question section, they were asked to answer the questions via the following way. “*Please answer the following questions. They serve the sole purpose of ensuring your understanding of the instructions.*"

**Questions Block 1:**

Question 1: A group consists of three participants: Participant A, Participant B, and Participant C. The roles within a group are randomly assigned.

Question 2: All group members first make decisions as if they were in the role of participant B before they learn what role they were randomly assigned to.

Question 3: All three situations in which decisions are made are payoff-relevant.

Question 4: Only the decision of the group member who was assigned the role of Participant B influences the payments of the group members in the randomly determined situation.

**Questions Block 2:**

Imagine that participant A is of type N.

Question 1.1: Which number of services maximizes the probability for Participant A of receiving 90 Taler?

Question 2.1: How high is the payment of participant B when he/she provides the optimal number of services for participant A?

Question 3.1: At which number of provided services is the probability to receive 90 Taler for Participant A at only 5%?

In addition, imagine that participant B informs the participant C that participant A is of type H.

Question 4.1: Which budget group is available to participant B? Budget Package I or Budget Package II?

Question 5.1: How high is the payment for Participant C if Participant A receives 3 units of services from Participant B?

Question 6.1: How high is the probability in percent that participant A receives 90 Taler if participant B provides him with 3 units of services?

Imagine that participant A is of type M.

Question 1.2: Which number of services maximizes the probability for Participant A of receiving 90 Taler?

Question 2.2: How high is the payment of participant B when she provides the optimal number of services for participant A?

Question 3.2:At which number of provided services is the probability to receive 90 Taler for Participant A at only 5%?

In addition, imagine that participant B informs participant C that participant A is of type M.

Question 4.2: Which budget package is available to participant B? Budget Package I or Budget Package II?

Question 5.2: How high is the payment for Participant C if Participant A receives 2 units of services from Paticipant B?

Question 6.2: How high is the probability in percent that Participant A receives 90 Taler if Participant B provides him/her with 2 units of services?

Imagine that participant A is of type H.

Question 1.3: Which number of services maximizes the probability for Participant A of receiving 90 Taler?

Question 2.3: How high is the payment of participant B when she provides the optimal number of services for participant A?

Question 3.3: At which number of provided services is the probability to receive 90 Taler for Participant A at only 5%?

In addition, imagine that participant B informs the Participant C that Participant A is of type N.

Question 4.3: Which budget package is available to participant B? Budget Package I or Budget Package II

Question 5.3: How high is the payment for Participant C if Participant A receives 1 unit of services from Paticipant B?

Question 6.3: How high is the probability in percent that participant A receives 90 Taler if participant B provides him with 1 units of services?

## Appendix F. Control Questions Medical Framing

The subjects had to answer questions among two blocks of answer categories. In the first block, the possible answers were either “*right*” or “*wrong*”. If a subject clicked on “*wrong*”, although “*right*” would have been correct they were informed: “*Your answer is not correct. Please change your entry.*” This information also appeared if a subject provided a wrong answer in the second question block of open questions. Subjects were asked to raise their hand, whenever they had a question. At the beginning of the control question section, they were asked to answer the questions via the following way. “*Please answer the following questions. They serve the sole purpose of ensuring your understanding of the instructions.*”

**Questions Block 1:**

Question 1: A group consists of three participants: Patient, Physician, and Health Insurance. The roles within a group are randomly assigned.

Question 2: All group members first make decisions as if they were in the role of the Physician before they learn what role they were randomly assigned to.

Question 3: All three situations in which decisions are made are payoff-relevant.

Question 4: Only the decision of the group member who was assigned the role of the Physician influences the payments of the group members in the randomly determined situation.

**Questions Block 2:**

Imagine that her Patient is of type N.

Question 1.1: Which number of Medical Services maximizes the probability for the Patient of receiving 90 Taler?

Question 2.1: How high is the payment of the Physician when she provides the optimal number of Medical Services for the Patient?

Question 3.1: At which number of provided Medical Services is the probability to receive 90 Taler for the Patient at only 5%?

In addition, imagine that the Physician informs the health insurance that the Patient is of type H.

Question 4.1: Which budget package is available to the Physician? Budget Package I or Budget Package II?

Question 5.1: How high is the payment for the Health Insurance if the Patient receives 3 units of Medical Services from the Physician?

Question 6.1: How high is the probability in percent that the Patient receives 90 Taler if the Physician provides him with 3 units of Medical Services?

Imagine that her Patient is of type M.

Question 1.2: Which number of Medical Services maximizes the probability for the Patient of receiving 90 Taler?

Question 2.2: How high is the payment of the Physician when she provides the optimal number of Medical Services for the Patient?

Question 3.2: At which number of provided Medical Services is the probability to receive 90 Taler for the Patient at only 5%?

In addition, imagine that the Physician informs the health insurance that the Patient is of type M.

Question 4.2: Which budget package is available to the Physician? Budget Package I or Budget Package II

Question 5.2: How high is the payment for the Health Insurance if the Patient receives 2 units of Medical Services from the Physician?

Question 6.2: How high is the probability in percent that the Patient receives 90 Taler if the Physician provides him with 2 units of Medical Services?

Imagine that her Patient is of type H.

Question 1.3: Which number of Medical Services maximizes the probability for the Patient of receiving 90 Taler?

Question 2.3: How high is the payment of the Physician when she provides the optimal number of Medical Services for the Patient?

Question 3.3: At which number of provided Medical Services is the probability to receive 90 Taler for the Patient at only 5%?

In addition, imagine that the Physician informs the health insurance that the Patient is of type N.

Question 4.3: Which budget package is available to the Physician? Budget Package I or Budget Package II?

Question 5.3: How high is the payment for the Health Insurance if the Patient receives 1 unit of Medical Services from the Physician?

Question 6.3: How high is the probability in percent that the Patient receives 90 Taler if the Physician provides him with 1 unit Medical Services?
